# Atmospheric-Pressure Mass Spectrometry by Single-Mode Nanoelectromechanical Systems

**DOI:** 10.1021/acs.nanolett.3c02343

**Published:** 2023-09-08

**Authors:** Batuhan E. Kaynak, Mohammed Alkhaled, Enise Kartal, Cenk Yanik, M. Selim Hanay

**Affiliations:** † Department of Mechanical Engineering, Bilkent University, 06800 Ankara, Turkey; ‡ UNAM - Institute of Materials Science and Nanotechnology, Bilkent University, 06800 Ankara, Turkey; § SUNUM, Sabancı University Nanotechnology Research and Application Center, 34956 Istanbul, Turkey

**Keywords:** nanoelectromechanical systems, single-mode sensing, NEMS mass spectrometry, atmospheric-pressure mass spectrometry, nanoparticle mass measurement, paddle NEMS

## Abstract

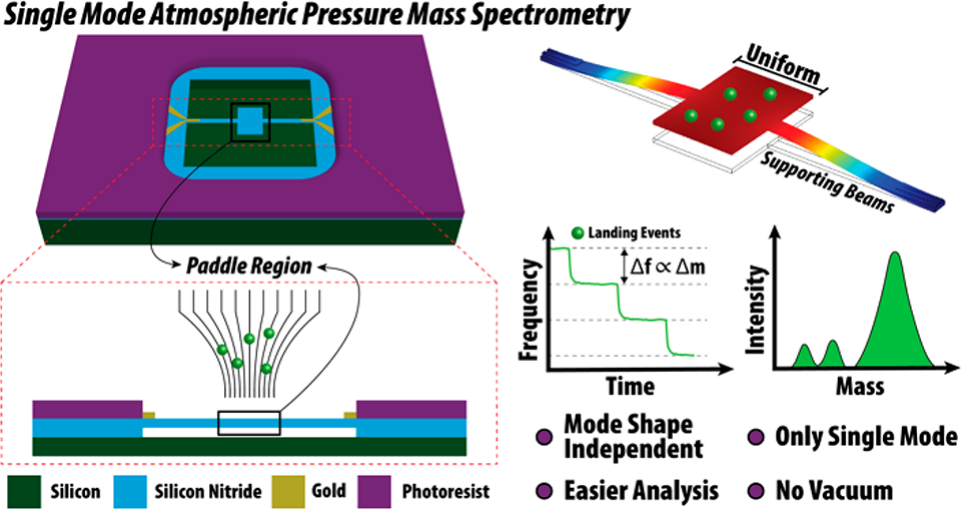

Weighing particles above the megadalton mass range has been a persistent challenge in commercial mass spectrometry. Recently, nanoelectromechanical systems-based mass spectrometry (NEMS-MS) has shown remarkable performance in this mass range, especially with the advance of performing mass spectrometry under entirely atmospheric conditions. This advance reduces the overall complexity and cost while increasing the limit of detection. However, this technique required the tracking of two mechanical modes and the accurate knowledge of mode shapes that may deviate from their ideal values, especially due to air damping. Here, we used a NEMS architecture with a central platform, which enables the calculation of mass by single-mode measurements. Experiments were conducted using polystyrene and gold nanoparticles to demonstrate the successful acquisition of mass spectra using a single mode with an improved areal capture efficiency. This advance represents a step forward in NEMS-MS, bringing it closer to becoming a practical application for the mass sensing of nanoparticles.

Nanoelectromechanical systems (NEMS) have proven their use in the mass spectrometry field^1−19^ for almost two decades, especially for analytes with masses that cannot be reached by conventional mass spectrometry techniques, i.e., >10 MDa, due to large mass-to-charge ratios (*m*/*z*).^20−22^ Therefore, the ability to measure these high mass values allows NEMS mass spectrometry (NEMS-MS) to be a potent tool for the characterization of metallic, ceramic, polymeric, and biological nanoparticles, e.g., exosomes, viruses, and lipid vesicles. Our recent study^16^ enabled the NEMS-MS technique to work under entirely atmospheric conditions, thus opening possibilities for enhancing the NEMS-MS technique while resolving major problems such as low capture efficiencies and high system costs. This technique does not suffer from bulky vacuum elements, leading to a lower system cost, and increases the capture efficiencies when compared with those of the systems that are deployed inside vacuum systems owing to the implementation of the polymeric focusing lens.

Typical device architectures in the NEMS-MS field have been doubly clamped beams, or cantilevers,^23,24^ with the notable exception of the use of membranes for higher collection efficiencies.^17,19^ In devices with beam architectures, two or more mechanical modes are needed to resolve the landing position and mass of each particle. Apart from requiring two measurement channels running in parallel, the applicability of multimode techniques^25^ requires the knowledge of mode shapes that deviate from their ideal values due to non-idealities in nanofabrication, the local stress on the device, and the random accumulation of adsorbates on the surface. In addition to these factors, a recent study^26^ uncovered that the displacement profiles of the NEMS devices are altered to some degree when the NEMS are operated under atmospheric conditions due to the higher dissipation^27−31^ (i.e., lower quality factor) caused by viscous damping of air compared to the vacuum, when the devices are driven from one extremity of the device.^32−35^ This difference is evident in flexural resonance modes, which are needed for accurate reverse calculation of the mass and the landing position of an analyte particle. Therefore, the governing equations for multimodal detection now introduce uncertainties for obtaining the mass spectrum of the particles. This alteration in the displacement profiles creates the need for devices that will directly relate the frequency shifts caused by a landing event to the mass of the device, while being independent of the mechanical mode shapes in the governing equations. Indeed, motivated in part by these considerations, recent studies with optomechanical sensors under vacuum conditions utilized a platform device with a uniform displacement field to measure, and hence, a single mechanical mode shape of the device was used.^15^

In this work, we have investigated the performance of a different class of NEMS architecture, paddle NEMS devices, for NEMS-MS under atmospheric conditions. The paddle NEMS device has a platform section in the active region to exhibit a uniform mode shape compared to conventional architectures reported in the literature, such as doubly clamped beams. This uniform mode shape enables us to calculate the mass of the landing analytes on the platform independent of the mode shape; rather, frequency shifts can be directly related to the mass of the device (for particles that land on the central platform). Therefore, the need for the use of higher mechanical modes and reverse-calculation equations that depend on mode shapes, which can cause further uncertainties for atmospheric operation, is eliminated. Moreover, the central placement and large platform area increase the unit-area collection efficiency by 4 times when compared with the doubly clamped beams.

Throughout the study, we operated our custom-built NEMS-MS system entirely under atmospheric conditions, including the electrospray ionization front end that generates isolated nanoparticles and the NEMS chip that contains the NEMS mass sensor and integrated, self-biasing ion lens.^16^ We first detected fluorescent nanoparticles using the paddle NEMS device and investigated these measurements by observing the same particles with fluorescence microscopy. In this case, we electrosprayed 200 nm fluorescent polystyrene nanoparticles (F-PSNP) on the NEMS device, and after collecting several such events, we classified the location of the nanoparticles and related their locations with the observed frequency shifts. In this way, we validated that for events landing on the central platform, the response of the NEMS is almost uniform.

After the validation of the mass sensing experiments with a uniform mode shape, we tested the performance of the paddle NEMS device in sensing by spraying a large number of 40 nm gold nanoparticles (GNPs). In this run, we observed a peak in the inferred diameter histogram that corresponds to a mean value of 43 nm, which again shows that the system is capable of measuring nanoparticles using only one mechanical mode in air. Therefore, the paddle NEMS devices decrease the electronic requirement to a single PLL circuitry while eliminating the uncertainties originating from the deviation of the displacement profiles from their ideal values (i.e., mode shapes).

Paddle NEMS devices are suspended structures made of LPCVD-grown, low-stress silicon nitride on a silicon substrate (Figure 1a). The fabrication of the device consists of electron beam lithography and photolithography techniques, along with dry etching to suspend the device. The supporting beam structures are typically 4 μm long and 400 nm wide on both sides, and the platform is 3 μm wide and 2 μm long. The fabrication flow is the same with a doubly clamped beam,^36,37^ including the integration step of the photoresist window.^16^ The implementation of the photoresist window facilitates the delivery of ions to the device surface (Figure 1a,b). The actuation and detection electrodes are U-shaped resistances that enable thermoelastic actuation and piezoresistive detection (Figure 1b).^36−38^

**Figure 1:**
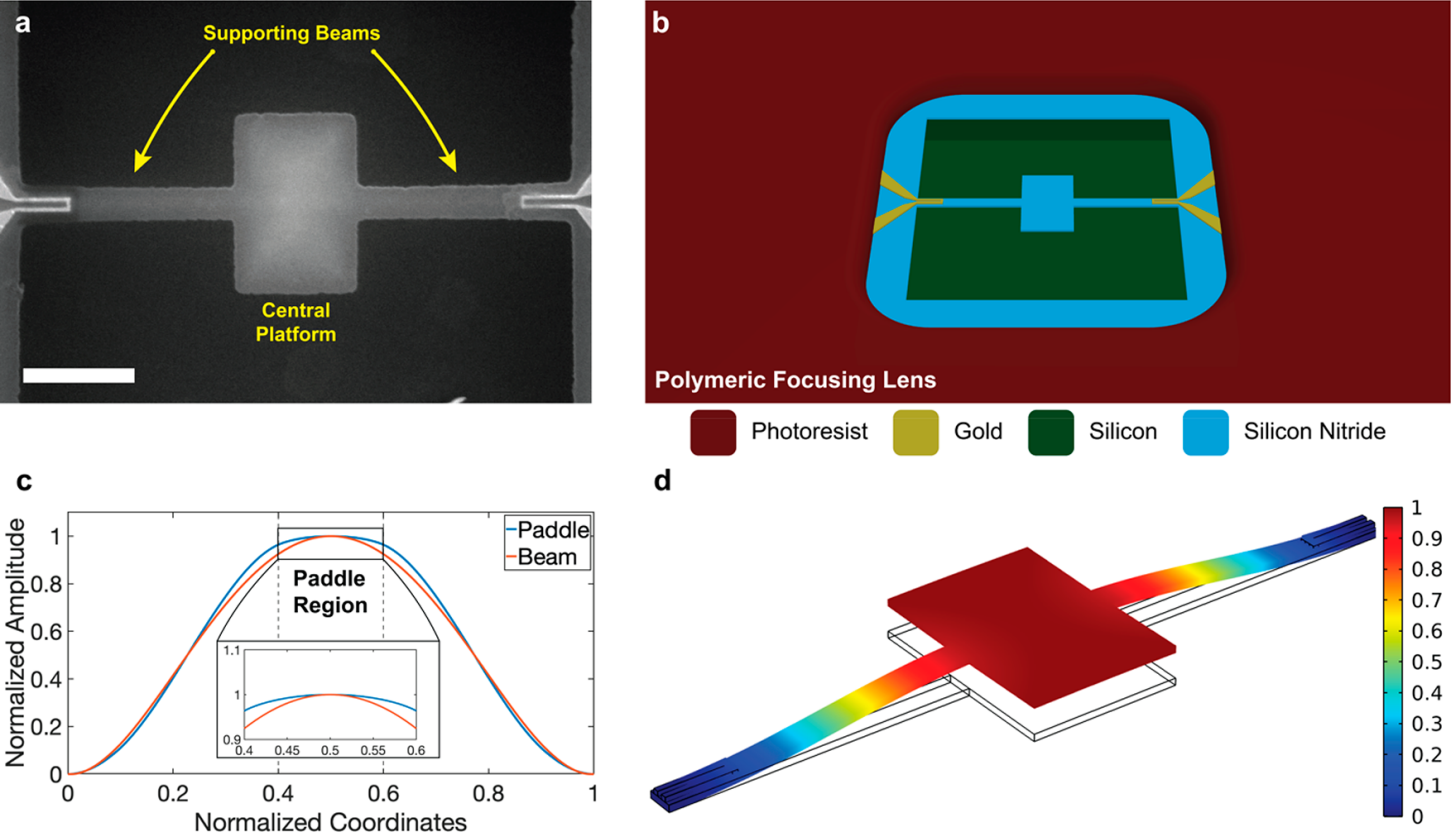
Device architecture and mode shape simulations. (a) SEM image featuring a paddle NEMS device with a central platform and supporting beams to connect the device to the anchor points. The scale bar is 2 μm. (b) Rendering of the device illustrating different layers on the substrate. (c) Mode shape of the paddle NEMS device compared to a normal doubly clamped beam. (d) First out-of-plane mode shape (*f* = 5.57 MHz) of the paddle NEMS device. The color map corresponds to the normalized displacement field.

In the typical doubly clamped beam architecture, the mode shape of the fundamental mechanical mode resembles a half-wavelength sine wave with an anti-node at the middle of the device (Figure 1c). For the paddle NEMS device, numerical simulations indicate that the central platform has a uniform mode shape with a maximum of a 5.2% difference between the extrema (Figure 1c,d). Over the entire platform, the standard deviation of the normalized displacement is ∼1% (Figure 1d). The use of the platform not only makes the mode shape uniform across a large area of the sensor but also increases the total sensor area. Indeed, the central platform comprises a large portion of the active sensor, ∼65% of the sensor’s total area, which is also the main region intended to detect the nanoparticles. However, the supporting beams on both sides are also responsive to the particles adsorbing on them; this situation currently introduces uncertainty into mass determination for the proposed technique.

Because the frequency shift caused by a particle is a function of the position in the case of doubly clamped beams, it is necessary to track the first two out-of-plane mechanical modes to resolve the landing position and the mass of the particle. However, in paddle NEMS devices, owing to the uniform mode shape on the platform, the frequency shift is a function of the device mass only, assuming the particle lands on the platform. Accordingly, we tracked only the first mechanical mode and observed the performance of the device in this setting.

We first conducted experiments with 200 nm F-PSNP to validate the uniformity of the mode shape on the platform of the paddle NEMS devices. The NEMS chip was placed in front of a custom-made ESI setup, which generates ionized nanoparticles that are then transported to the NEMS sensor with the help of the on-chip ion lens. In this experiment, we tracked the first mechanical mode because this mode features a uniform displacement profile across the middle platform, with an Allan deviation of 4.03 × 10^–6^ at the PLL time scale (set at 140 ms). The Allan deviation is only 3 times worse when compared to the doubly clamped beams operated under atmospheric conditions despite the increase in surface area (Figure S1). The minimum detectable mass of this device is 22.7 ag (13.7 MDa) under atmospheric conditions (effective mass calculations shown in section 4 of the Supporting Information).

In three consecutive sections, a total of eight particle landing events were detected in the PLL of the first mechanical mode (Figure 2a). The number of events was intentionally kept low to be able to relate the NEMS events to the microscopy images^39^ (attaining low event rates was facilitated by having two devices in one large focusing window). From the simulations, the particles landing at the platform are expected to induce frequency shifts of similar magnitudes. We indeed observed very similar values for frequency shifts for six of eight landing events. The microscope image of the tracked device can be seen in Figure 2b, and the fluorescent microscope image of the device after the last experiment can be seen in Figure 2c. From the fluorescent microscope image in Figure 2c, it is clear that indeed there are eight particles collected on the device, with six of them on the platform region (there are also additional particles close to the clamping points, but they generate much smaller frequency shifts). Out of the six events, one of them is located right at the edge intersection of the platform with the supporting beam. Table 1 lists these six landing events that are similar in terms of frequency shifts.

**Figure 2:**
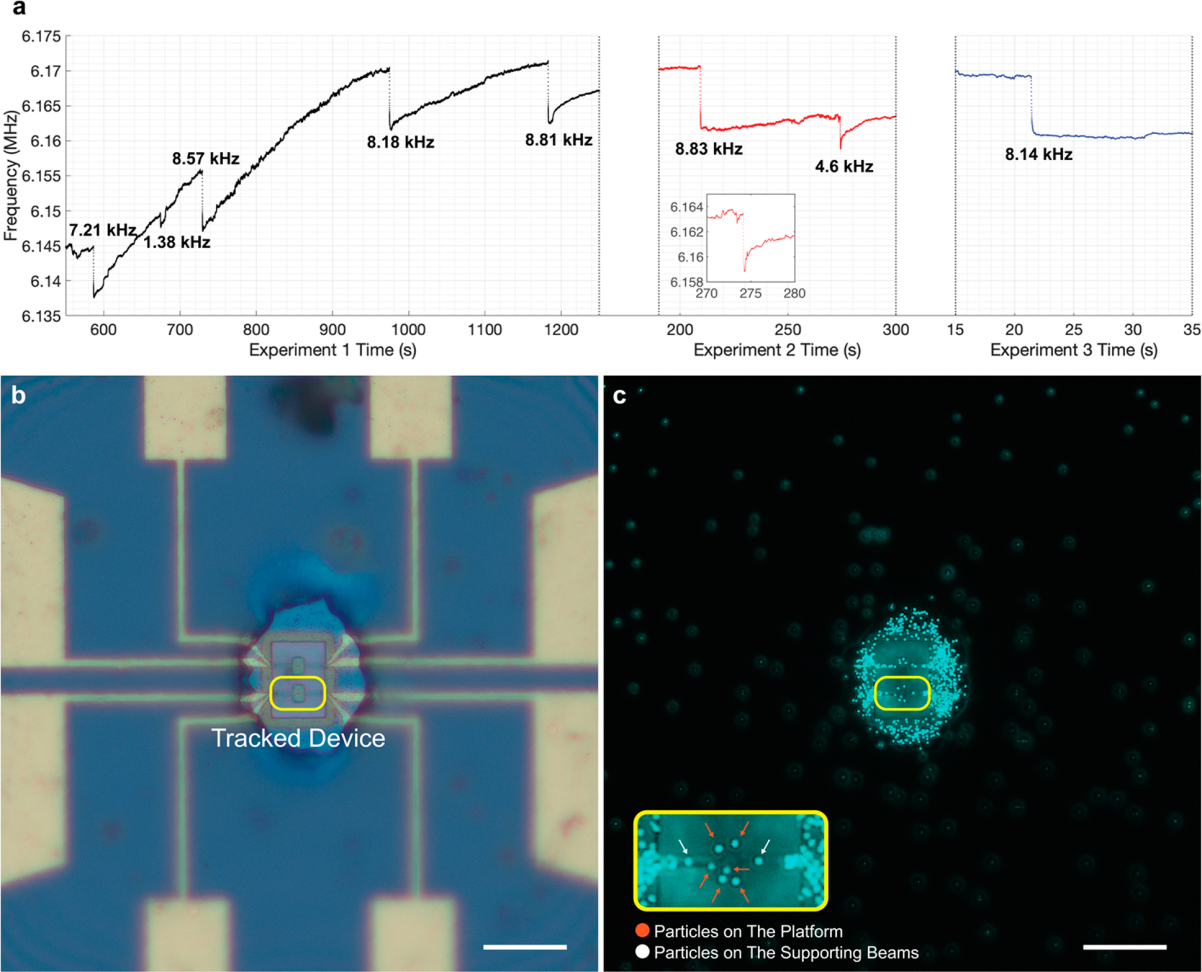
Uniform mode shape validation experiments for paddle NEMS devices. (a) PLL data of three consecutive experiments showing eight landing events on the tracked device. The rapid and partial recovery for the 4.6 kHz event (shown in the inset with more detail) is attributed to the desorption of a solvent shell around the nanoparticle. (b) Microscope image of the device after the third experiment. The yellow frame indicates the tracked device. The scale bar is 15 μm. (c) Fluorescence image of the device after the third experiment, showing six single 200 nm fluorescent polystyrene nanoparticles on the platform of the device (where the mode shape is uniform) and two on the supporting beams. The focusing capability of the device is evident by comparing the density of particles inside the window vs the density of those outside. The scale bar is 15 μm. The inset shows a close-up of the tracked device with arrows indicating the landing positions of the particles.

**Table 1:** Detailed Analysis of the Landing Events Shown in Figure 2a^a^

	event one	event two	event three	event four	event five	event six
frequency shift (Hz)	8140	8567	8176	8808	8831	7212
inferred diameter (nm)	212.1	215.9	212.4	217.7	217.9	204.0
mean (Hz)	8289					
standard deviation (Hz)	606					
coefficient of variation (%)	7.3					

a The coefficient of variation is 3.9% if the last event with a significantly lower frequency is omitted; this event is deemed to occur at the edge of the platform.

The landing events that are on the platform have a mean frequency shift of 8289 Hz with a standard deviation of 606 Hz. The coefficient of variation was calculated as 7.3% for the six landing events, which is smaller than the reported value of the polydispersity of the nanoparticles by the vendor (reported as <10% for the diameter). In agreement with the NEMS measurements, we can see in Figure 2c that six of the particles came to different locations on the platform, strongly indicating that these six particles correspond to the set of six frequency shifts with similar values in Table 1. We also observed that one of the frequency shifts (the last entry in Table 1) is smaller than the other five. We attribute this frequency shift to the particle on the left edge of the platform, where the displacement is smaller (Figure 2c, inset). If we were to exclude this event from the statistics, we would calculate a coefficient of variation of only 3.9%. The frequency shifts in the experiments, the fluorescent images, and the low coefficient of variation between events further validate that the mode shape is uniform on the platform for paddle NEMS devices. The coefficient of variation observed in Table 1 (7.3%) is larger than the coefficient of variation expected from the variation of responsivity (i.e., the square of the mode shape) on the platform (2.2%). We attribute this difference to the inherent polydispersity of the F-PSNPs (<10% for diameter). Thus, any landing event on the platform with the same-sized particles creates frequency shifts with similar dispersion levels. The two remaining events, with smaller values of 1.38 and 4.6 kHz, can be attributed to the two particles that can be seen on each side of the supporting beams (Figure 2c, inset).

In the next set of experiments, we increased the number of 200 nm fluorescent polystyrene nanoparticles collected on the paddle NEMS device by fabricating a NEMS chip with a single NEMS inside the focusing window, which was narrower and encapsulated the device more tightly. The Allan deviation was calculated as 2.75 × 10^–6^ (see Figure S2) for this device (Figure 3d), which corresponds to a minimum detectable mass of 20 ag (12 MDa) for particles landing on the platform (see the Supporting Information), which is comparable to the doubly clamped beam resonators used in the literature under atmospheric conditions.

**Figure 3:**
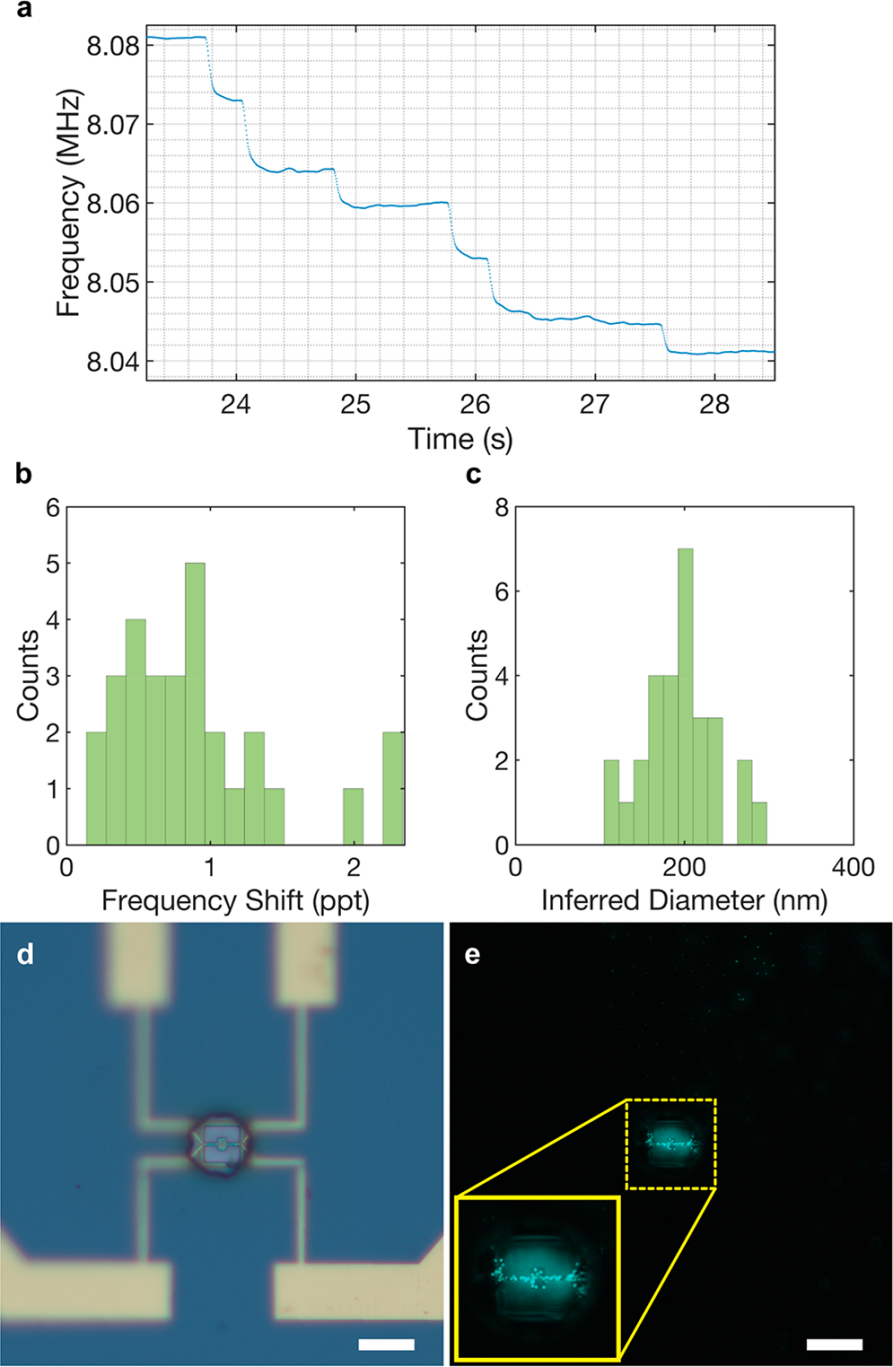
Paddle NEMS device used for mass spectrum experiments with 200 nm F-PSNP. (a) Section of the PLL data showing six consequent landing events. (b) Frequency shift histogram in parts per thousand (ppt). (c) Inferred diameter histograms constructed from all 29 landing events. The bin size is 17.5 nm (d) Bright-field and (e) fluorescent images of the device after the experiment. Scale bars are 15 μm for both images. The inset in panel e shows a close-up fluorescent image of the device in which the single particles on the middle platform can be observed.

In the experiment, the paddle NEMS device captured 29 landing events, again to ensure that NEMS measurements can be related to microscopy images. A section of the PLL where there are six landing events is shown in Figure 3a. From these 29 landing events, we constructed the frequency shift histogram, which can be seen in Figure 3b. We observed a peak at the expected frequency shift value of 200 nm F-PSNP in the histogram (the mean value of the frequency shifts is 8.55 × 10^–4^). Also, we observed more events in the lower-frequency shift range compared with the higher-frequency shifts, and we attribute this to the particles that landed on the supporting beams. The particles landing on the supporting beams are expected to create lower frequency-shifts when compared to the particles on the platform due to the lower displacement, thereby introducing uncertainty for the final mass spectrum (see Figure 1d). After acquiring the frequency shift values, we converted these values to mass values (Supporting Information) first, and then, by assuming particles as perfect spheres and using the density of polystyrene (1.05 g/cm^3^), we converted the mass values to obtain a histogram of inferred diameter values (Figure 3c). In the inferred diameter histogram (Figure 3c), we observed a peak at the expected 200 nm level, with almost half of the spectrum (14 events) accumulated at or adjacent to the 200 nm histogram bin (because the two adjacent bins are still within the specified range reported by the vendor). Furthermore, the particles with inferred diameters larger than the expected range are attributed to the particles that come as multiples due to the agglomeration in the solution and the ESI process, which was previously observed in the literature.^40^

After the experiment, we took both bright-field and fluorescent images of the device, which can be seen in panels d and e, respectively, of Figure 3. In the fluorescent image, we observed 13 particles on the platform, which correlates with the number of events in the inferred diameter histogram with 14 particles having diameters close to 200 nm (Figure 3c). Also, the histogram resulted in an average diameter of 195.5 nm, which is close to the expected value. The results show that paddle NEMS devices can perform mode-shape independent mass sensing experiments under atmospheric conditions.

The device in Figure 3 contains one paddle NEMS device inside a lensing window. The sensitive paddle area is located at the center of this window, and its large area can collect particles more efficiently than a doubly clamped beam, as expected of a two-dimensional geometry.^17,19^ The capture efficiency per unit area was observed to improve by a factor of 4 compared to that of the earlier polystyrene experiments with doubly clamped beams (Table S1).^16^ In the calculation of the capture efficiency, only the particles landing on the central platform were included (i.e., 14 events).

After validating the uniform mode shape on the platform for paddle NEMS devices, we tested a 40 nm gold nanoparticle sample (Nanopartz A11-40). After taking the open loop sweep, we configured the PLLs and calculated the Allan deviation as 6.35 × 10^–6^ (Figure S5) and the minimum detectable mass values as 40.3 ag (24.3 MDa).

Next, we electrosprayed the 40 nm GNP solution onto the NEMS chip (with a concentration of 8.53 × 10^9^ particles/mL). A section of the PLL data is shown in Figure 4a. Throughout the measurement, 139 events were collected. In the frequency shift histogram (Figure 4b), we observed a peak around the expected normalized frequency shift for a 40 nm GNP (1.44 × 10^–4^), which we attribute to the particle events that we have detected on the platform. The histogram’s lower end suggests particles landing on the supporting beams, which results in shifts lower than the expected mean value, due to the smaller displacement at the landing position.

**Figure 4:**
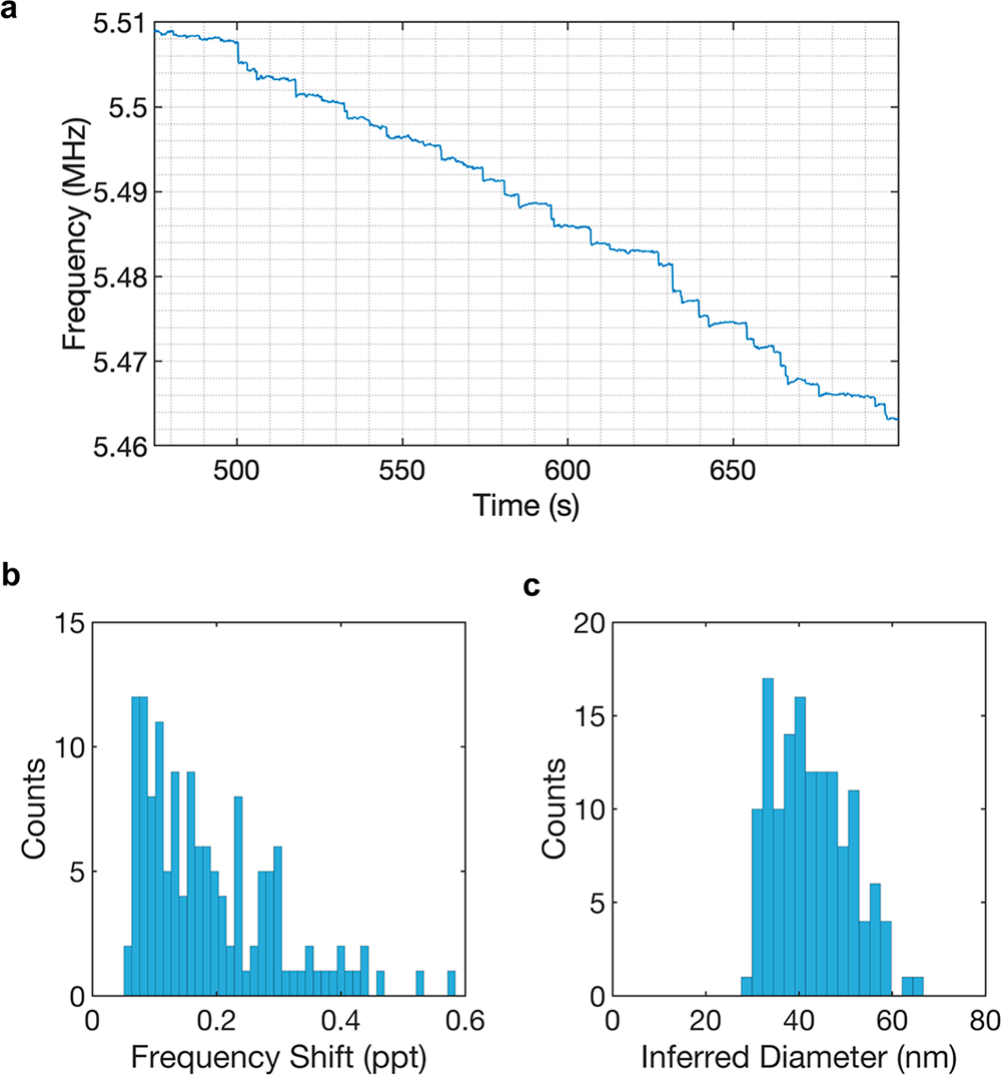
Measurements of 40 nm gold nanoparticle using a paddle NEMS device. (a) PLL frequency tracking while spraying the 40 nm gold nanoparticles. (b) Frequency shift histogram and (c) inferred diameter histogram, which was calculated on the basis of the calculated mass values from the frequency shifts using the density of gold (19.27 g/cm^3^).

The frequency shifts were converted into mass values and inferred diameter values using the density of gold (19.27 g/cm^3^) in Figure 4c, where events fall around 40 nm. The average value for the diameter calculation yielded ∼43 nm, which is close to the nominal value of 40 nm. Therefore, the results show that this device successfully detected 40 nm GNPs that landed on the middle platform. However, a portion of the events mostly corresponds to lower diameter values due to the particles landing on the supporting beams.

The peaks in the inferred diameter histograms of the 200 nm F-PSNP experiments (Table 1 and Figure 3c) as well as those in GNP experiments (Figure 4c) indicate that the paddle NEMS devices can characterize single-component nanoparticle samples using only one mechanical mode. Also, the calculations are now independent of mode shape; i.e., the change in mass is directly proportional to the frequency shift. Therefore, the alteration in displacement in the atmosphere can be omitted for the particles landing on the platform owing to the uniformity of mode shape independence. The events landing on the supporting beams constitute a limitation for these devices; however, for the analysis of samples composed of single species, mean values of the mass spectrum can be obtained.

The paddle NEMS architecture allowed us to have a large region with a uniform displacement profile on the NEMS device. Therefore, this uniformity allowed us to bypass the corrections required due to the alteration in the displacement profiles due to various effects, including the attenuation of mechanical waves under atmospheric conditions. We have shown this uniformity in the mode shape by spraying 200 nm F-PSNP onto the paddle NEMS device and correlated these measurements with microscopy images. In the mode shape validation experiments, we showed that the particles landing on the platform created similar frequency shifts. The landing events on the supporting beams created lower-frequency shifts due to the smaller displacement values on their landing positions. Moreover, the presence of a central platform in paddle NEMS devices increased the capture efficiency by a factor of 4. Finally, we performed a 40 nm GNP sensing experiment while PLL tracking, where a mean diameter of 43 nm was obtained from the diameter histogram. It is important to note that we observed landing events on the supporting beams in each experiment that resulted in inaccurate measurement results due to the smaller displacement on the supporting beams; this issue can be solved by the implementation of alternative lensing structures to further focus the particles on the central platform or by geometrical optimization of the paddle NEMS device, e.g., by reducing the supporting beam width. The paddle NEMS architecture provides a practical solution to the alteration in the displacement profile issue that affects atmospheric-pressure NEMS-MS by directly relating the frequency shifts to the particle mass.

## Supporting Information Available

Detailed discussion of the device fabrication, electrospray ionization (ESI) system, and sample preparation in addition to figures detailing the Allan deviation measurements and device modeling, such as the calculation of the effective mass (PDF)
