# Supplementary material for: Atmospheric-Pressure Mass Spectrometry by Single-Mode Nanoelectromechanical Systems
Source: Nano Lett. 2023 Sep 8;23(18):8553–9. doi: 10.1021/acs.nanolett.3c02343 (PMC10540252; doi:10.1021/acs.nanolett.3c02343)
Supplement: Supplementary file 1 — nl3c02343_si_001.pdf [file nl3c02343_si_001.pdf]

Supporting Information for

Atmospheric Pressure Mass Spectrometry by Single-Mode Nanoelectromechanical Systems

Batuhan E. Kaynak[1,2,‡], Mohammed Alkhaled[1,2,‡], Enise Kartal[1,2], Cenk Yanik[3], M. Selim Hanay [1,2,]*

[1] Department of Mechanical Engineering, Bilkent University, 06800 Ankara Turkey

[2] UNAM — Institute of Materials Science and Nanotechnology, Bilkent University, 06800 Ankara Turkey

[3] Sabancı University, SUNUM Nanotechnology Research and Application Center, 34956 Istanbul Turkey

[‡] These authors contributed equally.

[*] Corresponding author: selimhanay@bilkent.edu.tr

Table of Contents

S1. METHODS/EXPERIMENTAL ... 2

S2. ALLAN DEVIATION PLOT FOR THE PADDLE NEMS DEVICE USED FOR MODE SHAPE UNIFORMITY VALIDATION EXPERIMENTS USING FLUORESCENT POLYSTYRENE NANOPARTICLE ... 4

S3. ALLAN DEVIATION PLOT FOR THE PADDLE NEMS DEVICE USED FOR CALCULATING THE MASS SPECTRUM OF 200 NM FLUORESCENT POLYSTYRENE NANOPARTICLE ... 5

S4. EFFECTIVE MASS CALCULATIONS FOR PADDLE NEMS DEVICES ... 6

S5. ALLAN DEVIATION CALCULATION IN 40 NM GOLD NANOPARTICLE EXPERIMENTS ... 9

S6. TYPICAL FREQUENCY RESPONSE OF THE DEVICES ... 10

TABLE S1 : COLLECTION EFFICIENCY COMPARISON ... 11

REFERENCES: ... 12

S1. Methods/Experimental

Micro and Nanofabrication of Paddle NEMS Devices. The fabrication flow of the paddle NEMS devices starts with an EBL step using Poly (methyl methacrylate) (PMMA) on the 100 nm thick stoichiometric silicon nitride on a 500 µm thick silicon substrate (University Wafer 1917). The actuation and detection electrodes, alignment markers for the next steps, and contact pads are exposed and developed in this step. After the development, the chip was coated with a 5 nm chrome adhesion layer and a 70 nm gold layer using thermal evaporation. After the first step, one more EBL step takes place to define the paddle NEMS devices including the central platform and the connecting bridges. After the second EBL step and PMMA development, the chip was coated with 40 nm copper as a hard mask to protect the mechanical structures in the dry etching step. Next, the chip underwent two dry etching steps: the first step was the anisotropic etching of silicon nitride, and the second step was the isotropic etching of silicon. Isotropic etching of silicon suspended the paddle NEMS devices. After suspending the devices, the copper masking layer was wet-etched.

Lastly, the polymeric lens structure was implemented by photolithography. First, the device was coated using a thick photoresist (AZ 4533) to a thickness of 3.5 µm and then exposed to UV light through a copper mask to create openings in the active area and contact pads. After the exposure, the chip was developed and then wire bonded to a PCB to be used in the experiments.

Electrospray Ionization. The chip was placed in front of an ESI setup in order to facilitate the analyte delivery to the active area of the paddle NEMS device. The solution containing the nanoparticles was placed inside a glass syringe to deliver the solution to the online ESI tip. The ESI flow was then supplied with a high voltage (5.5 kV) to facilitate the ESI process. An extractor lens was placed after the ESI and was also kept at a high voltage (1.3 kV). The details of the ESI setup can be found in the reference.[16]

Gold Nanoparticle Sample Preparation. The solution that contains 40 nm nominal diameter particles in DI water was purchased from Nanopartz (A11-40). This stock solution was then diluted using an 80 mM Ammonium Acetate buffer to a final concentration of 8.53×10^{9} particles/mL, including the 10% v/v% methanol to facilitate the electrospray.

Fluorescent Polystyrene Nanoparticle Sample Preparation. The aqueous solution that contains the 200 nm fluorescent polystyrene particles was purchased from Thermo Scientific (Fluoro-Max Green Fluorescent Polymer Microspheres, CAT. NO: G200). This solution was diluted to a final concentration of 2.27×10^{10} particles/mL using 80 mM Ammonium acetate buffer and 10% v/v% methanol.

S2. Allan Deviation Plot for the Paddle NEMS Device Used for Mode Shape Uniformity Validation Experiments using Fluorescent Polystyrene Nanoparticle

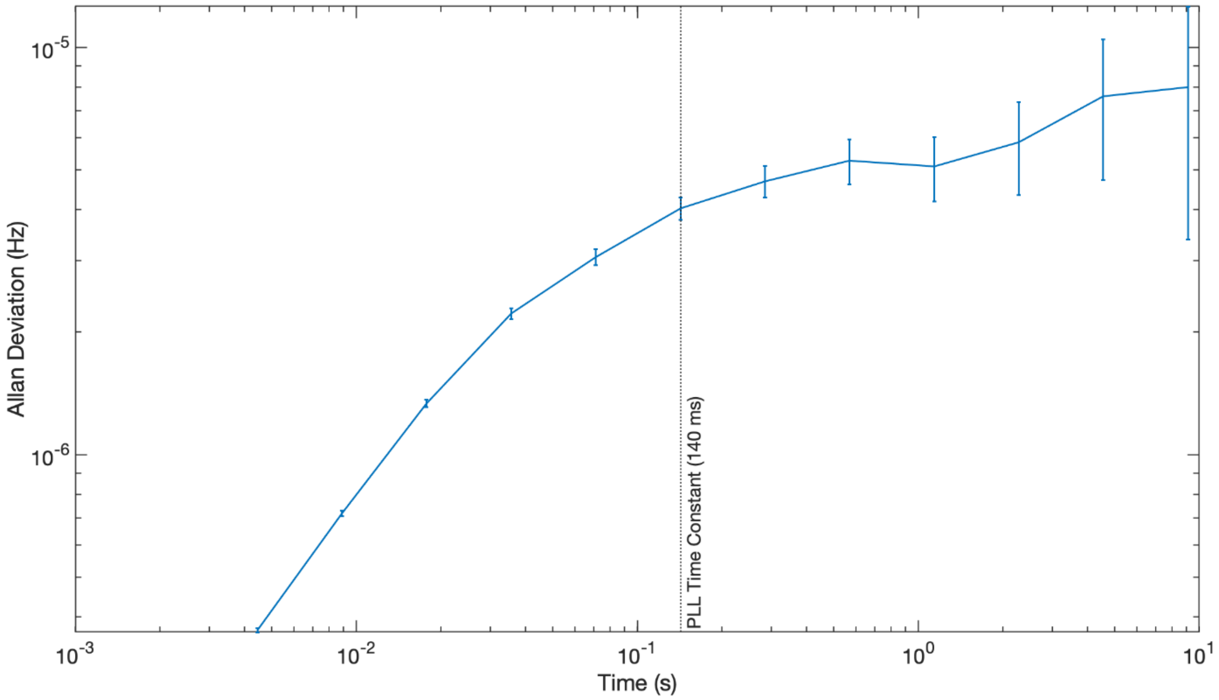

Supporting Figure S1. The Allan Deviation calculation for the paddle NEMS device used in the mode shape uniformity validation experiments with fluorescent nanoparticles. The Allan Deviation corresponds to the PLL time constant (140 ms) is $4.03{\times}10^{-6}$

S3. Allan Deviation Plot for the Paddle NEMS Device Used for Calculating the Mass Spectrum of 200 nm Fluorescent Polystyrene Nanoparticle

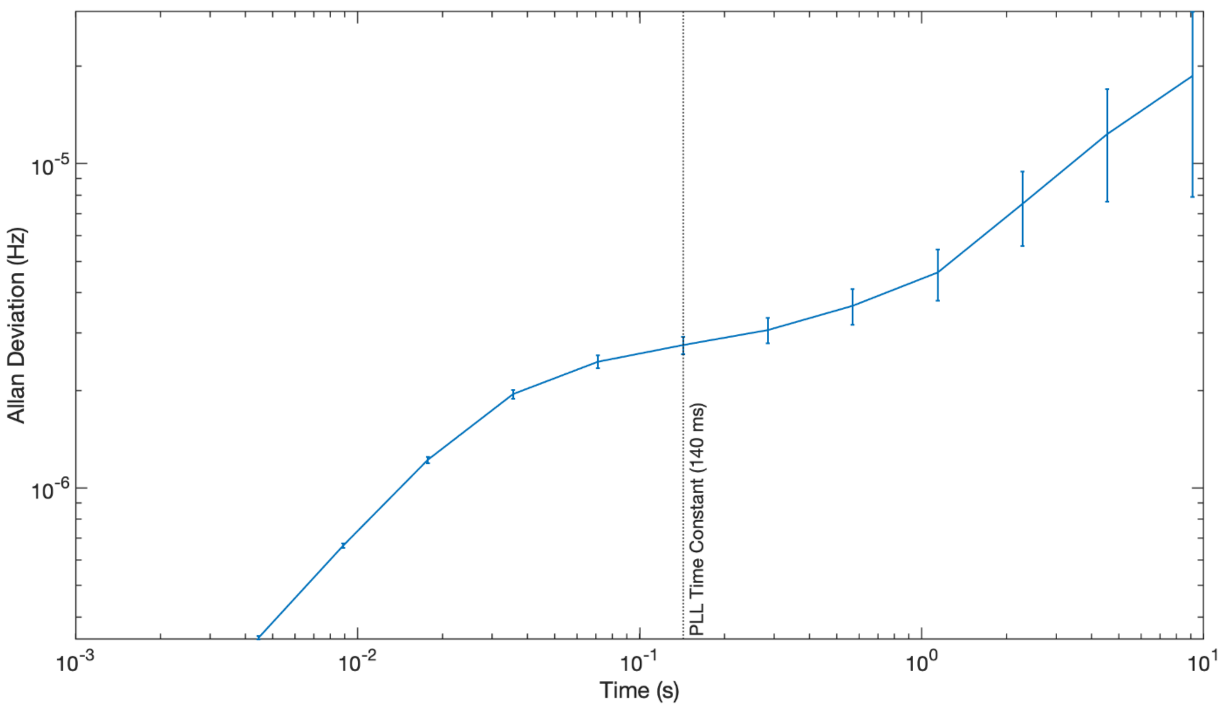

Supporting Figure S2. The Allan Deviation calculation for the paddle NEMS device used in experiments conducted to find the mass spectrum of the 200 nm F-PSNP. The Allan Deviation corresponds to the PLL time constant (140 ms) is 2.75×10^{-6}.

S4. Effective Mass Calculations for Paddle NEMS Devices

In order to calculate the mass of the particles, we need to calculate the effective mass of the paddle NEMS device, which accounts for the mode shape and the linear mass density of the device. The mode shape function of a paddle NEMS device can be seen below in Supporting Figure S3. The x-axis is normalized with respect to the device length, and the y-axis is normalized with respect to the maximum displacement. Also, the normalized linear mass density plot of the same paddle NEMS device can be seen in Supporting Figure S4.

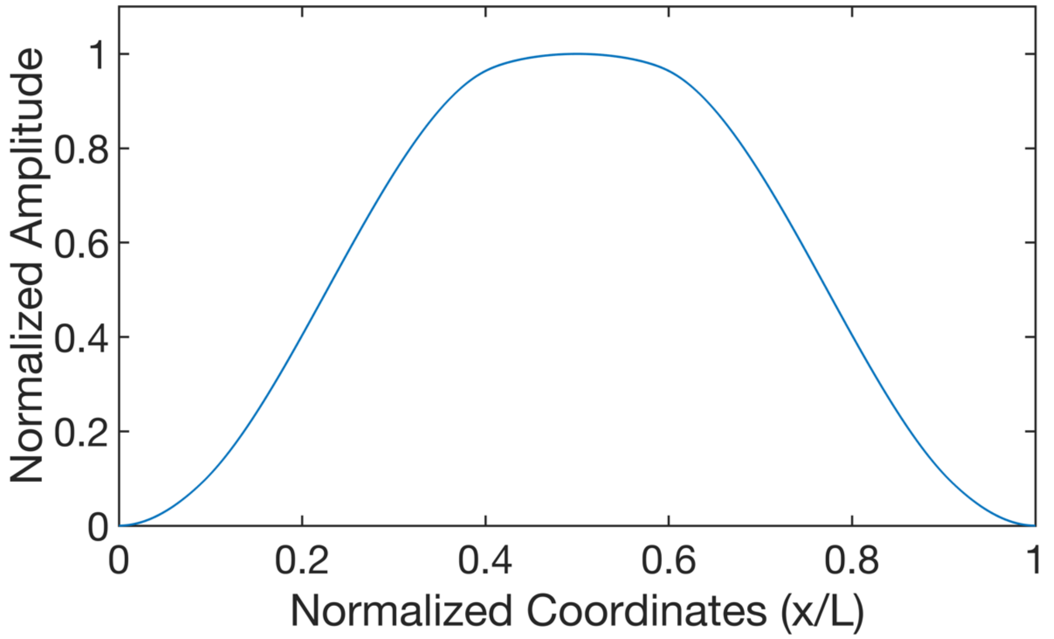

Supporting Figure S3. The mode shape function of a paddle NEMS device.

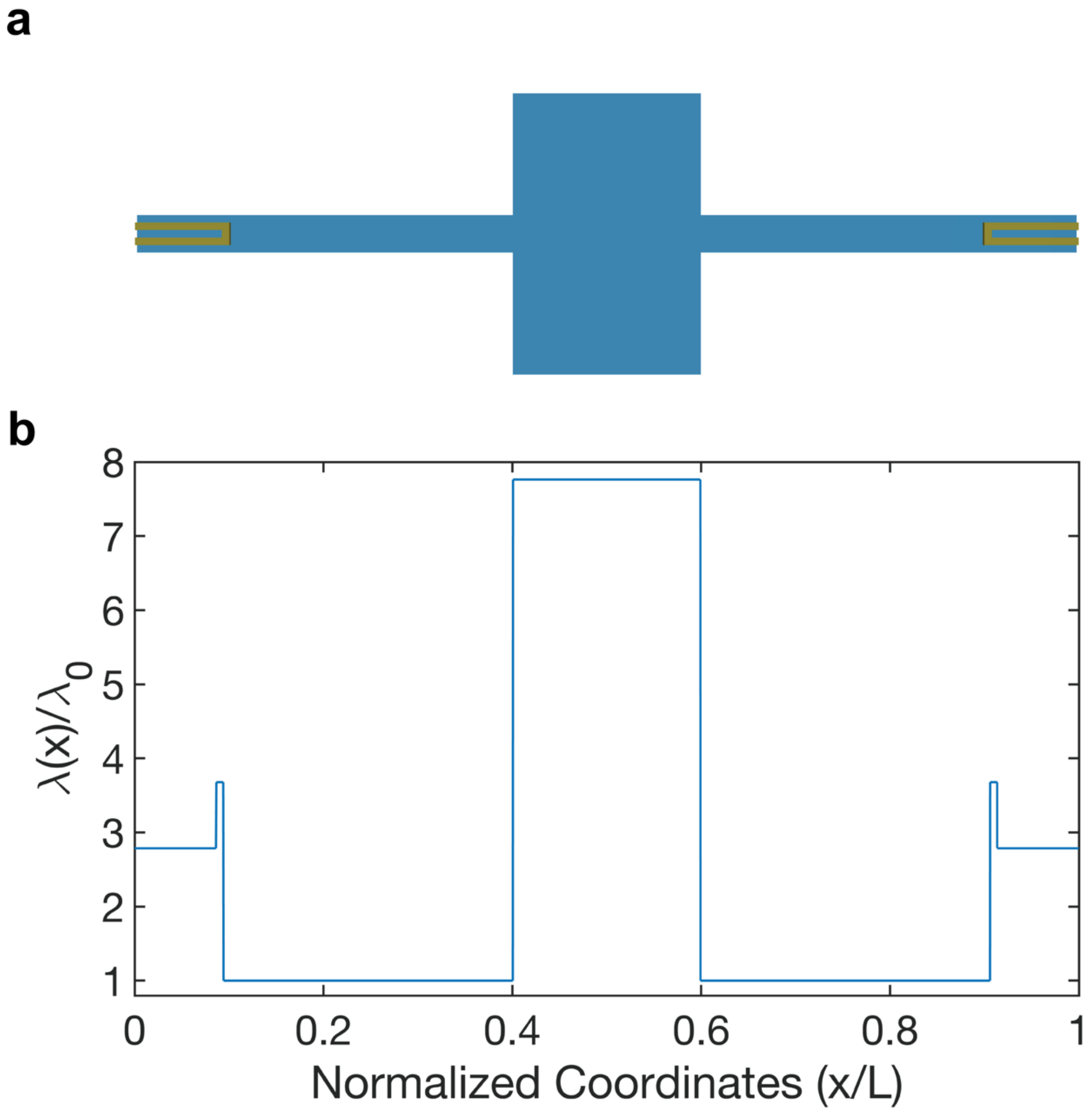

Supporting Figure S4. (a) CAD render of the paddle NEMS device. (b) The linear mass density function of the paddle NEMS device that is illustrated in panel (a).

Before calculating the mass resolution, we calculated the total masses for each device using the equation below:

$$M_{total} = \int_{x=0}^{x=L} \lambda(x) dx$$

where $\lambda(x)$ is the linear mass density function. The linear mass density function handles the device as 1D and accounts for the masses of additional structures on the device, such as the resistances and the platform. After calculating the total mass of the device, we calculate the effective mass of the device considering the mode shape using the equation below:

$$M_{eff} = \int_{x=0}^{x=L} \lambda(x)\phi(x)^2 dx$$

Using the equation above with the mode shape function and the linear mass density function, we can find the effective mass of the device.

In the case of a doubly-clamped NEMS device, the equation below is being used to calculate the landing position and the mass of the particles:

$$\Delta f_n = -\frac{f_n}{2M_{eff}} \Delta m \phi_n(x)^2$$

where Δf_n is the frequency shift at a landing event, f_n is the frequency of the tracked mechanical mode, Δm is the added particle mass and n denotes the mode number. Since the mode shape on the platform is uniform and we use only the fundamental mode, the equation above becomes:

$$\Delta m = -2M_{eff} \frac{\Delta f}{f}$$

The equation above allows us to find the mass of each landing particle on the platform without any dependence on the mode shape.

After establishing the equation for the calculation of the mass of the particles, we use the following equation for the calculation of the minimum detectable mass:

$$\delta m_{min} = \sqrt{2} \times 2M_{eff} \langle \frac{\Delta f}{f_0}_{min} \rangle$$

where $\langle\frac{\Delta f}{f_0}_{min}\rangle$ is the frequency stability of the device, *i.e.* Allan Deviation. Both the equation for the calculation of the landing particles' mass and the equation for the minimum detectable mass is used in the uniform mode shape validation and mass sensing experiments.

S5. Allan Deviation Calculation in 40 nm Gold Nanoparticle Experiments

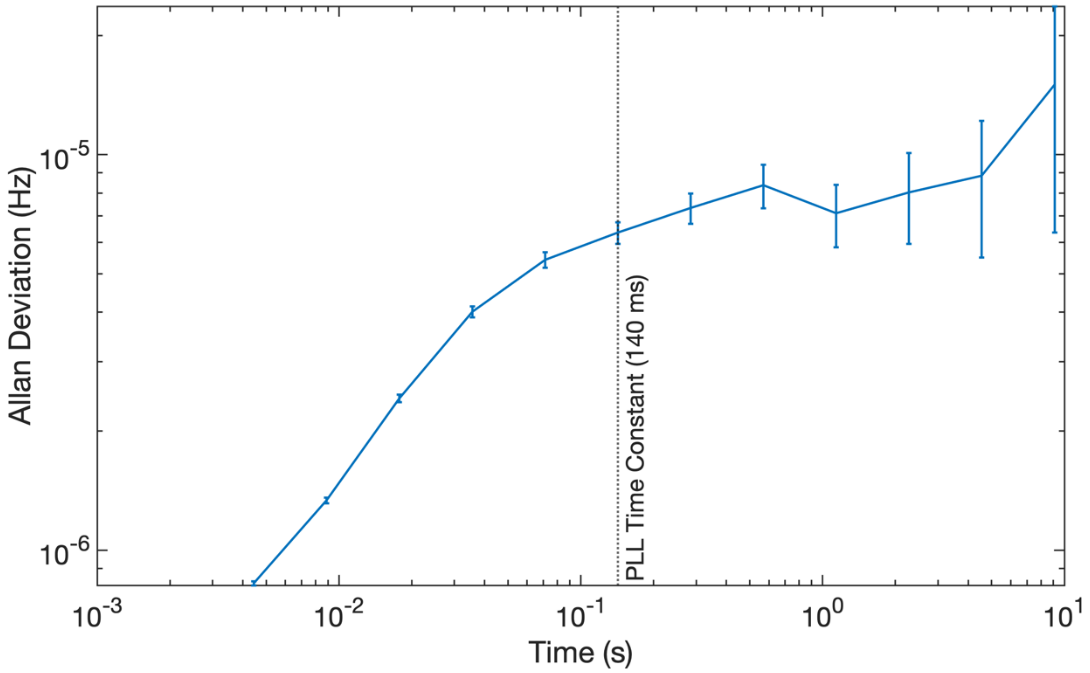

Supporting Figure S5. The Allan Deviation calculations for the device used in the 40 nm GNP experiments. The Allan Deviations was calculated as 6.35×10^{-6} at the PLL time constant, which is 140 ms.

S6. Typical Frequency Response of the Devices

The frequency spectrum of the device that we have used in our 40 nm GNP experiments below. The sweep is taken under atmospheric pressure (as in the sensing experiments). Only the fundamental mode is used in the experiments.

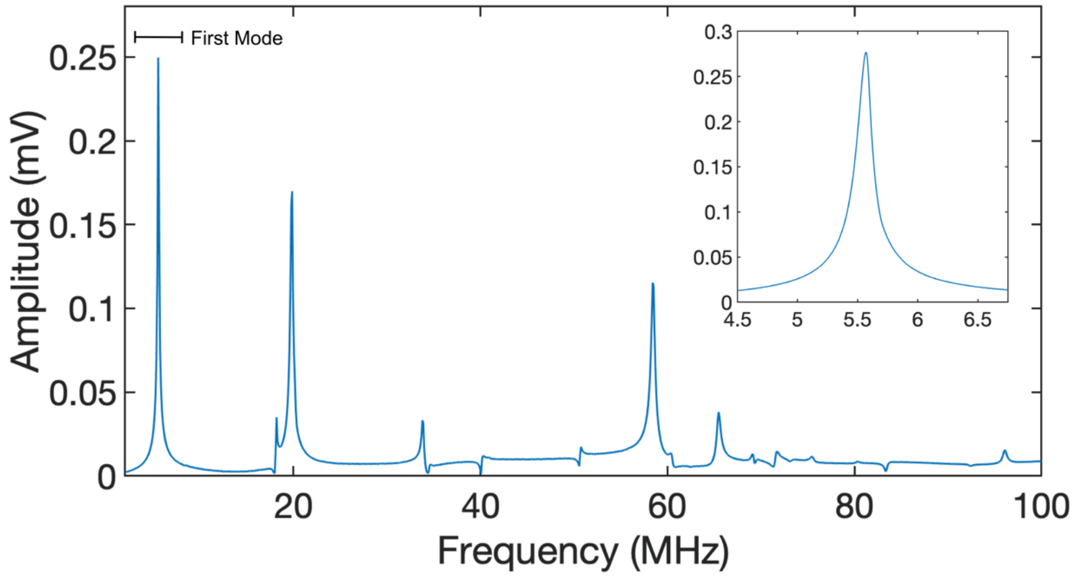

Supporting Figure S6. (a) The frequency spectrum of a typical paddle device operated in air, the inset shows the frequency sweep near the first mode which is used in the experiments.

Table S1 : Collection Efficiency Comparison

Ref.	Particle Type	Particle Diameter [nm]	Solution Particle Concentration [p/mL]	Detection Efficiency [Detected particles/Sprayed particle]	NEMS Capture Cross-section Area [μm^2]	Capture Efficiency per μm^2
**Erdogan et Al. 2022**[1]	100 nm f-PSNP	100	$3.55x10^9$	1 per 648 thousand	22.5	**1 per 14.6 million**
This Work	200 nm f-PSNP	200	$2.27x10^{10}$	1 per 261 thousand (all particles)	11.5 (total sensor area)	**1 per 3.0 million**
	200 nm f-PSNP	200	$2.27x10^{10}$	1 per 540 thousand (Particles with inferred diameters close to 200 nm)	7.1 (platform area)	**1 per 3.8 million**

References:

1. Erdogan, R. T.; Alkhaled, M.; Kaynak, B. E.; Alhmoud, H.; Pisheh, H. S.; Kelleci, M.; Karakurt, I.; Yanik, C.; Şen, Z. B. l.; Sari, B. Atmospheric Pressure Mass Spectrometry of Single Viruses and Nanoparticles by Nanoelectromechanical Systems. *ACS Nano* **2022,** 16, (3), 3821-3833.
